# Accuracy of the CMS Chronic Conditions Data Warehouse Algorithms in Medicare Advantage and Fee-For-Service Data

**DOI:** 10.1093/geroni/igaf122.4397

**Published:** 2025-12-31

**Authors:** Xiecheng Chen, Chan Mi Park, Lan Luo, Eunji G Kim, Ellen McCarthy, Dae Hyun Kim

**Affiliations:** Hebrew Rehabilitation Center, Roslindale, Massachusetts, United States; Hebrew SeniorLife, Harvard Medical school, Boston, Massachusetts, United States; Marcus Institute for Aging Research, Boston, Massachusetts, United States; Marcus Institute for Aging Research, Boston, Massachusetts, United States; Hebrew SeniorLife, Boston, Massachusetts, United States; Hebrew SeniorLife, Boston, Massachusetts, United States

## Abstract

Since over half of Medicare beneficiaries now enroll in Medicare Advantage (MA), and Chronic Condition Warehouse (CCW) algorithms are commonly used to identify chronic conditions from Medicare claims data, we evaluated whether their performance extends from fee-for-service (FFS) claims to MA encounter data using NHATS Round 9 (2019) as the reference. NHATS respondents with at least 24 months of continuous MA or FFS enrollment before the survey date were linked to Medicare claims and encounter files. CCW definitions with a two-year look-back were applied for conditions such as arthritis, chronic lung disease (COPD plus asthma), diabetes, heart disease (heart failure plus ischemic heart disease), hypertension, and osteoporosis. Self-reported, physician-diagnosed conditions served as the reference, with estimates weighted to the national survey. The differences between programs were tested using survey-weighted Wald tests. The sample included 1,708 MA and 2,239 FFS beneficiaries. High sensitivity was observed for diabetes and hypertension across both programs, with high specificity for diabetes (0.95 in MA vs. 0.92 in FFS) and moderate specificity for hypertension (0.70 in MA vs. 0.68 in FFS). Chronic lung disease demonstrated moderate sensitivity (0.64–0.66) and high specificity (0.90–0.91), with minimal differences between MA and FFS. Heart disease showed moderate sensitivity and specificity for both programs. Arthritis and osteoporosis had lower sensitivity (0.50 in MA vs. 0.54 in FFS) for arthritis, but were highly specific (0.85 in MA vs. 0.85 in FFS). Overall, CCW algorithms for these common conditions performed comparably in MA and FFS, and the observed differences between programs were small.

